# Editorial: Immunomodulatory factors, conversion, and postoperative adjuvant therapy for hepatobiliary tumors based on immunotherapy

**DOI:** 10.3389/fimmu.2023.1218845

**Published:** 2023-05-23

**Authors:** Er-lei Zhang

**Affiliations:** Hepatic Surgery Center, Tongji Hospital, Tongji Medical College, Huazhong University of Science and Technology, Wuhan, China

**Keywords:** hepatocellular carcinoma, immunotherapy, targeted therapy, conversion therapy, postoperative adjuvant therapy

Hepatocellular carcinoma (HCC) ranks as the second leading cause of cancer death in China and worldwide (1). Surgical resection remains the most effective radical treatment method, but the resection rate is as low as 20%-30% in clinical practice due to late diagnosis and delayed treatment in most HCC patients, which contributes to the poor prognosis of HCC. Even with radical resection for early-stage HCC, the recurrence rate is still high, especially for those with microvascular invasion. How to prevent HCC recurrence is more important for long-term outcomes (2). Notably, improving HCC prognosis though surgical technique is unrealistic nowadays. Immunotherapy has ushered in a new era in the systemic treatment of advanced HCC in the past few years (3). Immunotherapy may manipulate the immune system to recognize and attack tumor cells and has displayed promising clinical effects in the treatment of advanced HCC. However, the low objective response rate and therapy resistance still represent major challenges for immunotherapy. There is an urgent need to find biomarkers to predict the efficacy of immunotherapy and targeted therapy and to screen out the sensitive population. Furthermore, conversion therapies are advocated to facilitate subsequent resection for selected patients with technically unresectable BCLC stage A HCC or technically resectable BCLC stage C HCC in China (4). The current Research Topic mainly focuses on the critical role of immunotherapy in the treatment of HCC and investigates the therapeutic predictors of immunotherapy. In addition, it also elucidates the immunomodulatory effects of tyrosine kinase inhibitors on immunotherapy and identifies the appropriate patients, which may provide promising strategies in targeted therapy and immunotherapy of HCC.

HCC recurs in 70–80% of cases following potentially curative treatments, and the immune microenvironment of the liver plays a key role in tumor recurrence. There is no proven postoperative adjuvant therapy for HCC after curative resection. The time to HCC recurrence after curative resection is an important prognostic factor; early recurrence appears to be associated with poorer prognosis compared with those with late recurrence. Therefore, how to reduce early recurrence is of great importance for prolonging the overall survival time for HCC patients following curative resection. Previous studies indicated that microvascular invasion (MVI) is an independent risk factor for early recurrence (5). A recent randomized, open-label, multicenter trial indicated that postoperative adjuvant hepatic arterial infusion chemotherapy (HAIC) with 5-fluorouracil and oxaliplatin (FOLFOX) significantly improved the disease-free survival (DFS) benefits with acceptable toxicities in HCC patients with MVI (the median DFS was 20.3 months in the treatment group versus 10.0 months in the control group) (6). An ongoing phase III trial (IMbrave 050) evaluated the efficacy of atezolizumab plus bevacizumab in high-risk HCC after curative resection or ablation, and the preliminary results are positive (7). More evidence is needed on the strategies of postoperative adjuvant treatments in the future in order to shed new light on ways to improve the surgical prognosis of HCC patients.

More than half of HCC patients are diagnosed with advanced stages in China, of which macrovascular invasion (MaVI) is one of the most common signs. The main type of MVI is portal vein tumor thrombus (PVTT), and the incidence in HCC patients is 44%–62.2% (8). Nearly all the international guidelines recommend non-surgical treatments, including transarterial chemoembolization (TACE) or systemic therapy, as the first-line treatment for HCC patients with PVTT (BCLC stage C and CNLC stage IIIa). However, quite a few HCC patients with MaVI can undergo surgical resection and achieve superior survival results compared with non-surgical treatments. Under the circumstances, the Chinese HCC guideline recommends that surgical resection should be considered if the PVTT can be completely removed during the operation, followed by postoperative local or systemic therapy to prevent recurrence (4). Systemic therapies such as tyrosine kinase inhibitors (TKIs) plus programmed death-1 (PD-1) inhibitors and locoregional treatments including TACE, HAIC, and radiation have been widely recommended for the treatment of those HCC patients with advanced stages to achieve survival benefit. These comforting results of survival benefit have been associated with relatively satisfactory objective response rates reflecting a significant reduction in tumor burden. Due to these advances in systemic treatments,a hot discussion has been prompted regarding their role in conversion therapy prior to liver resection. HAIC, TACE, and radiation therapy are also candidates for conversion therapy in advanced HCC or unresectable HCC and are currently gaining increasing attention as good combination methods with immunotherapy due to their role in modulating the tumor microenvironment. Previous studies reported that the conversion resection rate based on these combination therapies ranged from 20% to 50% (4). Nonetheless, the long-term survival benefit for liver resection after conversion therapy remains to be further evaluated in the future.

Not all HCC patients can benefit from immunotherapy and targeted therapy. A considerable number of HCC patients displays primary resistance to immunotherapy and might benefit from antiangiogenics, either alone or in combination with immunotherapy. The reported objective response rates based on immunotherapy range from 30% to 50% for HCC patients (4). Therefore, more and more studies are focusing on looking for predictive biomarkers for immunotherapy (9, 10). Programmed death ligand 1 (PD-L1) expression in HCC cells received the most attention in previous studies. However, PD-L1 expression alone has limited predictive value for immunotherapy in HCC. Thus, subsequent studies indicated that tumor mutational burden (TMB), tumor characteristics, and microsatellite status were independent predictive biomarkers (10). Despite tumor tissue being the preferred source in evaluating these characteristics, liquid biopsy may overcome the matter of unrepeatable nature for tissue assessment and track tumor changes, which is applied in some medical centers. Recently, tumor genetic phenotypes, tumor microenvironment features, gut microbiome, and systemic inflammation have all been potential predictors of response to immunotherapy, and they are awaiting further validation in the clinical setting.

Moreover, the immunomodulatory mechanisms of TKIs and local treatments on various immune cells and their synergistic effects for HCC treatment remain to be further elucidated. Identification of the effective predictors response to immunotherapy and reasonable selection of a combination therapy base on immunotherapy will facilitate treatment allocation at each stage of HCC. Perhaps in the near future, palliative liver resection for advanced HCC could become a reality.

## Author contributions

The author confirms being the sole contributor of this work and has approved it for publication.
